# *HLA-C* dysregulation as a possible mechanism of immune evasion in SARS-CoV-2 and other RNA-virus infections

**DOI:** 10.3389/fimmu.2022.1011829

**Published:** 2022-10-17

**Authors:** Eleonora Loi, Loredana Moi, Paola Cabras, Giulia Arduino, Giulia Costanzo, Stefano Del Giacco, Henry A. Erlich, Davide Firinu, Aldo Caddori, Patrizia Zavattari

**Affiliations:** ^1^ Department of Biomedical Sciences, Unit of Biology and Genetics, University of Cagliari, Cagliari, Italy; ^2^ Department of Internal Medicine, Hospital SS. Trinità, Cagliari, Italy; ^3^ Department of Medical Sciences and Public Health, University of Cagliari, Cagliari, Italy; ^4^ Department of Genetics and Genomics, Children’s Hospital Oakland Research Institute, Oakland, CA, United States

**Keywords:** COVID-19, SARS-CoV-2, DNA methylation, HLA-C, gene expression, enhancer transcriptional regulation, symptomatic and asymptomatic COVID-19, upper airways cells

## Abstract

One of the mechanisms by which viruses can evade the host’s immune system is to modify the host’s DNA methylation pattern. This work aims to investigate the DNA methylation and gene expression profile of COVID-19 patients, divided into symptomatic and asymptomatic, and healthy controls, focusing on genes involved in the immune response. In this study, changes in the methylome of COVID-19 patients’ upper airways cells, the first barrier against respiratory infections and the first cells presenting viral antigens, are shown for the first time. Our results showed alterations in the methylation pattern of genes encoding proteins implicated in the response against pathogens, in particular the *HLA-C* gene, also important for the T-cell mediated memory response. *HLA-C* expression significantly decreases in COVID-19 patients, especially in those with a more severe prognosis and without other possibly confounding co-morbidities. Moreover, our bionformatic analysis revealed that the identified methylation alteration overlaps with enhancers regulating *HLA-C* expression, suggesting an additional mechanism exploited by SARS-CoV-2 to inhibit this fundamental player in the host’s immune response. HLA-C could therefore represent both a prognostic marker and an excellent therapeutic target, also suggesting a preventive intervention that conjugate a virus-specific antigenic stimulation with an adjuvant increasing the T-cell mediated memory response.

## 1 Introduction

The present study is part of a project that aimed to compare the genomic DNA methylation profile of cells collected by nasopharyngeal swabs from symptomatic patients with severe acute respiratory syndrome coronavirus 2 (SARS-CoV-2) infection, responsible for the coronavirus disease 2019 (COVID-19), with that of comparable samples from subjects positive for SARS-CoV-2 but asymptomatic, from subjects negative for SARS-CoV-2 and from individuals with a previous SARS-CoV-2 infection. The epithelial respiratory cells represent the first barrier between the organism and the surrounding environment. They are known to act as antigen-presenting cells (APCs) during respiratory viral infections (1, 2).

This study is based on previous knowledge obtained on cells infected with another pathogenic coronavirus, such as MERS-CoV, of alterations in the methylome of the infected host cell. In particular, previous studies have shown that MERS-CoV targets regions of the host cell genome that are essential to trigger an immune response against pathogens such as bacteria and viruses, e.g. *HLA* (Human Leukocyte Antigens) genes, inhibiting them in this role and thus rendering the infected individual incapable, or much less efficient in triggering an effective immune response against infection (3).

*HLA* genes encode proteins essential in immune function (the Major Histocompatibility Antigens), involved in the presentation of the antigen to the immune system; they belong to two different types: HLA class I and class II antigens. HLA/MHC class I molecules (A, B, and C) present intracellular antigens, such as viral or tumor antigens, to CD8 positive T cells (cytotoxic T lymphocytes, CTLs), stimulating a cytotoxic immune response and to natural killer (NK) cells. For example, if the cell becomes infected with a virus, the HLA system carries protein fragments of the virus to the surface of the cell so that it can be destroyed by the immune system. These peptides, usually small polymers of 9 amino acids, are produced from proteins digested in proteasomes. CTLs can recognize HLA-peptide complex through their T cell receptor.

HLA/MHC class II (DP, DM, DOA, DOB, DQ and DR) present extracellular antigens, to CD4 lymphocytes, inducing a helper T cell response which consists of supporting the activation of CD8 lymphocytes and establishing long-term memory. In addition, helper T lymphocytes support the production by B lymphocytes of neutralizing antibodies against the specific antigen.

Viruses are intracellular antigens, and they can be subjected to proteolytic digestion in the proteasome. In the endoplasmic reticulum, these antigenic peptides bind HLA class I molecules. However, both class I and II HLA molecules can process intracellular and extracellular antigens. The HLA-peptide complexes are then translocated to the cell membrane, where class I are ubiquitously expressed, while class II are expressed by cells specialized for antigen presentation, such as dendritic cells, monocytes, macrophages and B lymphocytes.

As noted above, one of the mechanisms by which viruses modify the expression of immune-related genes in the host cell, including *HLA* genes, is to induce changes in the methylation profile of genomic DNA. DNA methylation, at cytidines adjacent to guanosines (CpG loci), is an epigenetic mechanism normally used in cells contributing to regulate gene expression. Typically, a methylation status of a gene’s CpGs in the regulatory sequences is associated with its shutdown, while the absence of methyl groups in those regions is typically associated with active transcription of that gene. Dysregulation of this crucial mechanism is involved in the development and progression of many human diseases (4), including cancer as the most noteworthy example in which the identification of DNA methylation alterations has also provide the definition of clinically-relevant biomarkers (5–11). In the context of infectious diseases, a growing body of evidence underlines its role in their pathogenesis and in the development of chronic diseases triggered by the modifications induced by the pathogens (12).

Since SARS-CoV-2 belongs to coronaviruses such as SARS-CoV-1 and MERS-CoV, we hypothesized that it may act similarly by influencing the expression of *HLA* genes by modulating their methylation profile.

Recently, the research group of Prof. Esteller conducted an epigenome-wide association study (EWAS) to identify candidate loci regulated by DNA methylation, potentially involved in the onset of COVID-19 in patients without comorbidities. Among the main findings of this study, whole blood DNA methylation alteration was observed in genes, including *HLA* class I C (*HLA-C*), mainly involved in the response of interferon to viral infection (13). It is also important to consider that *HLA-C*, the most recently evolved class I locus (only present in humans and great apes (14)), although expressed on the cell surface about ten times lower than *HLA-A* and *B*, represents a potentially particular target for the mechanisms put in place by viral infections, acting as a ligand for both T cell receptors and NK cell receptors (15, 16).

More recently, Balnis and colleagues published the results of a study in which they compared differentially methylated regions of circulating blood DNA from hospitalized COVID-19-positive and COVID-19-negative patients and previously reported data from healthy individuals collected before the pandemic. The authors also conducted an mRNA expression analysis of immuno-related genes, showing that DNA methylation alterations were inversely correlated with gene expression levels, confirming a prevalence of promoter hypermethylated profile in severe COVID-19 patients (17).

Our study investigated the levels of *HLA-C* expression in upper respiratory tract cells, showing that symptomatic patients show significantly lower levels than asymptomatic patients and SARS-CoV-2 negative people. These results are consistent with previous observations and contribute to our understanding of the role that these DNA methylation alterations may play in the pathological course of COVID-19.

**Figure 1:**
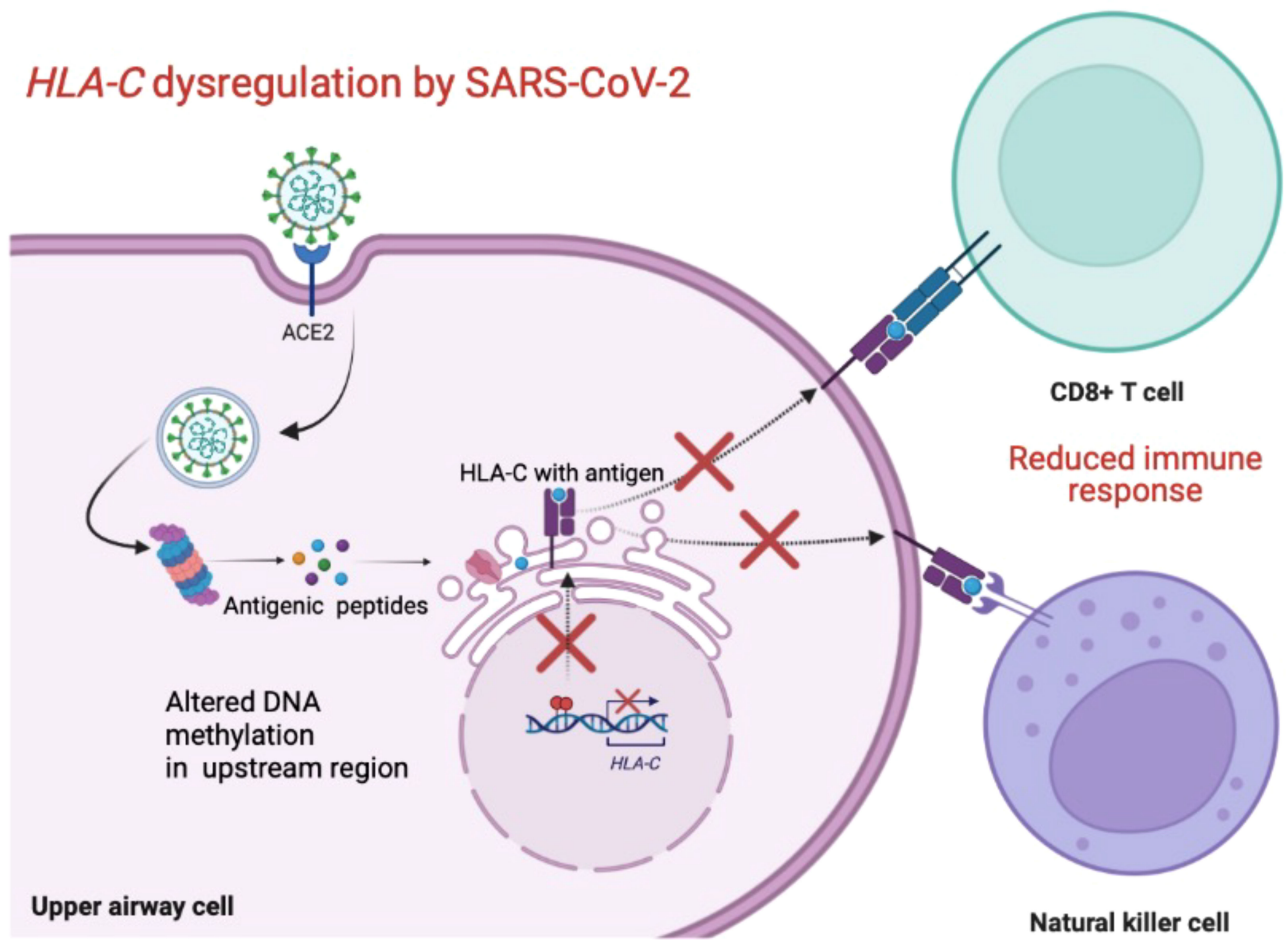
Schematic overview of the possible mechanism used by SARS-CoV-2 to downregulate HLA-C and evade the host’s immune response. Created with BioRender.com.

**Figure 1** shows an overview of possible mechanism used by SARS-CoV-2 to evade the host’s immune response.

## 2 Materials and methods

### 2.1 Study design and sample cohort

**Figure 2:**
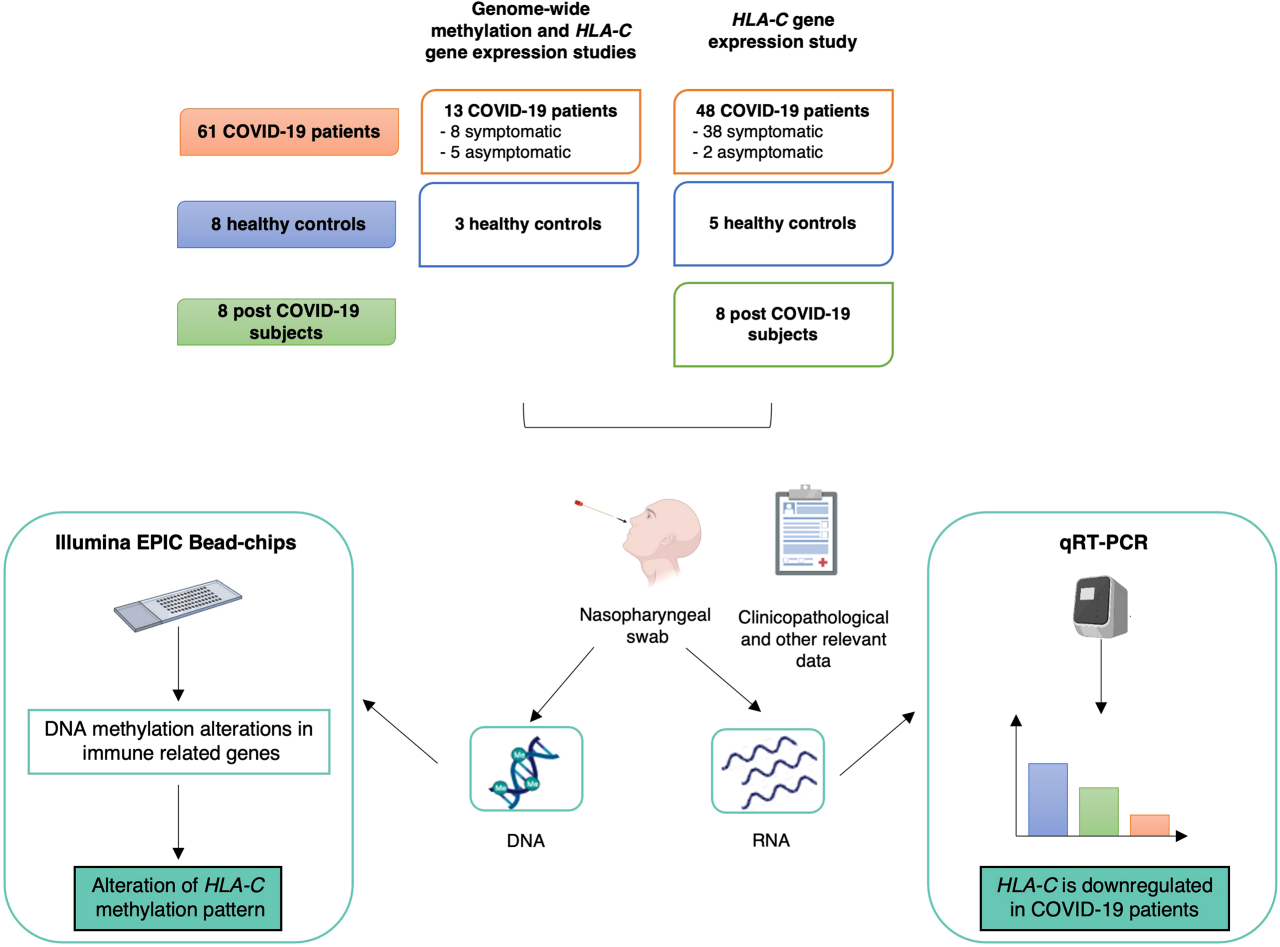
Study design, workflow and main results. The upper part describes the sample cohort used for the genome-wide methylation and *HLA-C* gene expression analyses. Below, workflow and main results are summarized. Created with BioRender.com.

Enrolment of the participants took place between May 2020 and April 2021. The study workflow is summarized in **Figure 2**.

#### 2.1.1 Sample cohort for the genome-wide methylation study

The genome-wide methylation study was performed on 13 COVID-19 patients and three healthy controls.

COVID-19 patients were recruited at “Santissima Trinità” hospital (Cagliari, Italy). Eligibility criteria for the COVID-19 group included: at least 18 years of age, positivity to nasopharyngeal swab for SARS-CoV-2 by PCR. Patients were classified as symptomatic (n=8), who were hospitalized for severe COVID-19 pneumonia (n=7) and/or showed other COVID-19 related symptoms, and as asymptomatic (n=5).

Healthy controls were healthcare workers from the University Hospital “Policlinico Duilio Casula” (Monserrato, Italy) resulted negative to screening for SARS-CoV-2.

Clinicopathological characteristics of the COVID-19 patients and relevant data of healthy controls are summarized in **Supplementary Table 1**.

#### 2.1.2 Sample cohort for the *HLA-C* gene expression study

*HLA-C* gene expression was tested in 61 COVID-19 patients (including the 13 patients subjected to the global DNA methylation profiling and 48 additional patients), eight healthy controls and eight subjects with a previous SARS-CoV-2 infection. COVID-19 patients were enrolled at “Santissima Trinità” (Cagliari, Italy) and “Policlinico Duilio Casula” (Monserrato, Italy) hospitals, post-COVID-19 participants were recruited from “Policlinico Duilio Casula”. COVID-19 patients (>18 years old and positive for SARS-CoV-2 infection) were symptomatic (n=45) and asymptomatic (n=8). Clinicopathological information were not available for eight samples.

Participants were eligible as post-COVID-19 subjects if they had a previous SARS-CoV-2 infection (5 symptomatic and 3 asymptomatic) and resulted negative to two consecutive (in a range of 2-3 days) SARS-CoV-2 screening tests by PCR. Of note, one subject was analysed before SARS-CoV-2 infection (T0) and at two time points post asymptomatic COVID-19: T1, coincident with the date of the first negative swab (last day of treatment with hydroxychloroquine) and T2, 18 days after.

SARS-CoV-2 test negative participants were healthcare workers from “Policlinico Duilio Casula”.

**Supplementary Table 1** reports clinicopathological characteristics of the COVID-19 patients and relevant data of post-COVID-19 and healthy subjects.

A further analysis was conducted by applying the same criteria applied in Castro de Moura et al. work (13) to exclude patients with age > 61 years and comorbidities (including obesity, diabetes, hypertension, autoimmune disorders, and chronic cardiovascular or lung diseases).

### 2.2 Sample collection and processing

Nasopharyngeal swabs were collected from the participants and immediately stored in a tube with TRIzol reagent (Thermo Fisher Scientific, Waltham, Massachusetts, USA).

#### 2.2.1 DNA and RNA extraction and quantification

After removing the swab, taking care to carefully squeeze all the mucus soaked in it, chloroform is added to TRIzol. After homogenization, different phases are separated: a clear upper aqueous layer (containing RNA), an interphase, and a lower organic layer (containing the DNA and proteins). The addition of isopropanol to the aqueous phase allows for the isolation of RNA by precipitation. Adding ethanol to the interphase/organic layer allows DNA to precipitate. The addition of isopropanol to the phenol-ethanol supernatant allows the proteins to precipitate. After washing to remove any impurities, DNA, RNA and proteins are resuspended in aqueous solutions and used for molecular investigations.

DNA and RNA concentration was quantified by UV spectrophotometry (NanoPhotometer™ Pearl, Implen) and by fluorometric reading (Quant-iT™ PicoGreen^®^ dsDNA Assay Kit; Quant-iT RiboGreen RNA Kit).

#### 2.2.2 Bisulfite conversion

DNA samples were treated with sodium bisulfite using EZ DNA Methylation Gold Kit (Zymo Research).

#### 2.2.3 Whole-genome methylation assay

Bisulfite converted samples were shipped to the “Italian Institute for Genomic Medicine” (Candiolo, Italy) and subjected to DNA methylation analysis using the Illumina Infinium MethylationEPIC BeadChips, which interrogate >850,000 loci. Following a rigorous quality control of the post-analysis data, the company communicated that all the samples analysed passed this control. Raw data were transmitted to the research group by Wetransfer.

#### 2.2.4 Gene expression assay

Reverse transcription of 1 μg (with the exception of few cases with limited RNA amount) of RNA to cDNA was performed using the High-Capacity Kit (Applied Biosystems, Carlsbad, CA, USA). Gene expression analysis of *HLA-C* (Hs03044135_m1) and *GAPDH* (Hs03929097_g1), used as endogenous gene, was performed by TaqMan Gene Expression Assays (FAM-MGB) (Thermo Fisher Scientific, Waltham, Massachusetts, USA). The assays were conducted in triplicate and the experiment was conducted on a DNA Engine Opticon 2 Real-Time Cycler (Bio-Rad, Hercules, CA, USA) using the following PCR conditions: initial activation 95°C for 10 minutes, 50 cycles of denaturation at 95°C for 15 seconds and annealing/extension at 60°C for 1 minute.

### 2.3 Genome-wide methylation data analysis

Raw DNA methylation data (idat files) were analysed using RnBeads (18, 19) installed in R environment. RnBeads workflow consists in: quality control, filtering and normalization, export of processed data, exploratory analysis and differential DNA methylation analysis. The background was subtracted using the methylumi package (method “enmix.oob”) (20). The methylation β values were normalized using the BMIQ normalization method (21). The differential methylation analysis was performed based on two comparisons: COVID-19 *vs* controls and asymptomatic *vs* symptomatic COVID-19 patients.

CGIs were annotated to the nearest genes and transcripts using R annotation package FDb.InfiniumMethylation.hg19 (22).

We focused our attention on differentially methylated CpG islands (CGI) (comb.pval < 0.05 and/or Δβ > 0.05 indicating hypermethylated CGI or Δβ < -0.05, indicating hypomethylated CGI) associated with immunologically relevant genes. The list of genes curated with functions and Gene Ontology terms was retrieved from Immport.org. The categories included: Antigen Processing and Presentation (148 genes), Antimicrobials (535 genes), BCR Signaling Pathway (272 genes), Chemokine Receptors (53 genes), Chemokines (102 genes), Cytokine Receptors (307 genes), Cytokines (456 genes), Interferon Receptor (3 genes), Interferons (17 genes), Interleukins (47 genes), Interleukins Receptor (42 genes), Natural Killer Cell Cytotoxicity (134 genes), TCR signaling Pathway (291 genes), TGFb Family Member (33 genes), TGFb Family Member Receptor (12 genes), TNF Family Members (12 genes), TNF Family Members Receptors (19 genes).

For *HLA-C* gene, the analysis was also conducted at the CpG site level.

### 2.4 Validation dataset

Processed Illumina EPIC methylation data of bisulfite converted DNA from whole blood of 102 COVID-19 patients and 26 non-COVID-19 patients (17) were retrieved from the NCBI Gene Expression Omnibus (GEO) Portal under the accession number GSE174818. Data were downloaded using the Bioconductor package “GEOquery” (23).

### 2.5 Bioinformatic enhancer analysis

We consulted HACER database (http://bioinfo.vanderbilt.edu/AE/HACER/index.html) to investigate the potential presence of integrated enhancers associated with *HLA-C* gene.

### 2.6 Statistics

#### 2.6.1 DNA methylation data

As reported above, DNA methylation data were analysed using RnBeads (18, 19). This tool performs the differential methylation analysis with hierarchical linear models as implemented in the limma package (24). Gender and age data were used as covariate for adjusting p-values in the limma differential methylation analysis. RnBeads computes p-values for all covered CpG sites. The uncorrected CpG-level p-values are then combined at the level of predefined genomic regions using a generalization of Fisher’s method (25). Aggregate p-values are subjected to multiple-testing correction using Bonferroni-Benjamini false discovery rate (FDR).

#### 2.6.2 Gene expression data

Gene expression data were analysed by the ΔΔCt method (26). Statistics was calculated using Welch’s t-test considering the average ΔCt for each tested group.

### 2.7 Ethics statement

The study was conducted in accordance with the Declaration of Helsinki, and the protocol was approved by the Ethics Committee of “ATS Sardegna” (224/2020/CE). All the analysed biological samples were obtained with written informed consent from participants prior to inclusion in the study.

## 3 Results

We performed a whole genome methylation profiling of 13 COVID-19 patients, divided into asymptomatic and symptomatic patients, and three healthy controls.

### 3.1 DNA methylation alterations of immune-related genes in COVID-19 patients

Based on the knowledge that the infection of other coronaviruses is associated with DNA methylation alteration of genes involved in the generation of immune responses against viruses and bacteria, we focused our attention on CGIs associated with immunologically relevant genes belonging to the categories reported in **Table 1**. The table shows the number of differentially methylated CGIs according to different criteria.

**Table 1:** Differentially methylated CpG islands in COVID-19 patients *vs* controls and asymptomatic *vs* symptomatic COVID-19 patients.

	COVID-19 *vs* controls			Asymptomatic *vs* symptomatic		
Category	Comb.p.val <0.05	Δβ ≥ 0.05	Δβ ≤ -0.05	Comb.p.val <0.05	Δβ ≥ 0.05	Δβ ≤ -0.05
Antigen Processing and Presentation	2 (hypomethylated)	1	7	0	2	1
Antimicrobials	5 (hypomethylated)	16	15	6 (hypermethylated)	11	5
BCR Signaling Pathway	2 (hypomethylated)	3	12	1 (hypermethylated)	2	4
Chemokine Receptors	0	0	2	1 (hypermethylated)	1	1
Chemokines	1 (hypomethylated)	3	2	2 (hypermethylated)	3	1
Cytokine Receptors	3 (hypomethylated)	20	15	8 (7 hypermethylated and 1 hypomethylated)	10	3
Cytokines	4 (hypomethylated)	9	17	3 (hypermethylated)	7	6
Interferon Receptor	0	0	0	0	0	0
Interferons	0	0	0	0	0	0
Interleukins Receptor	0	1	1	0	0	0
Interleukins	1 (hypomethylated)	0	1	0	0	0
Natural Killer Cell Cytotoxicity	4 (3 hypomethylated and 1 hypermethylated)	4	15	2 (hypermethylated)	3	2
TCR signaling Pathway	2 (hypomethylated)	2	13	1 (hypermethylated)	2	2
TGFb Family Member Receptor	0	0	0	1 (hypermethylated)	0	0
TGFb Family Member	0	2	3	0	0	0
TNF Family Members	0	0	0	0	0	0
TNF Family Members Receptors	0	0	0	0	1	0
**Total number of alterations**	24	61	103	25	42	25

**Figure 3:**
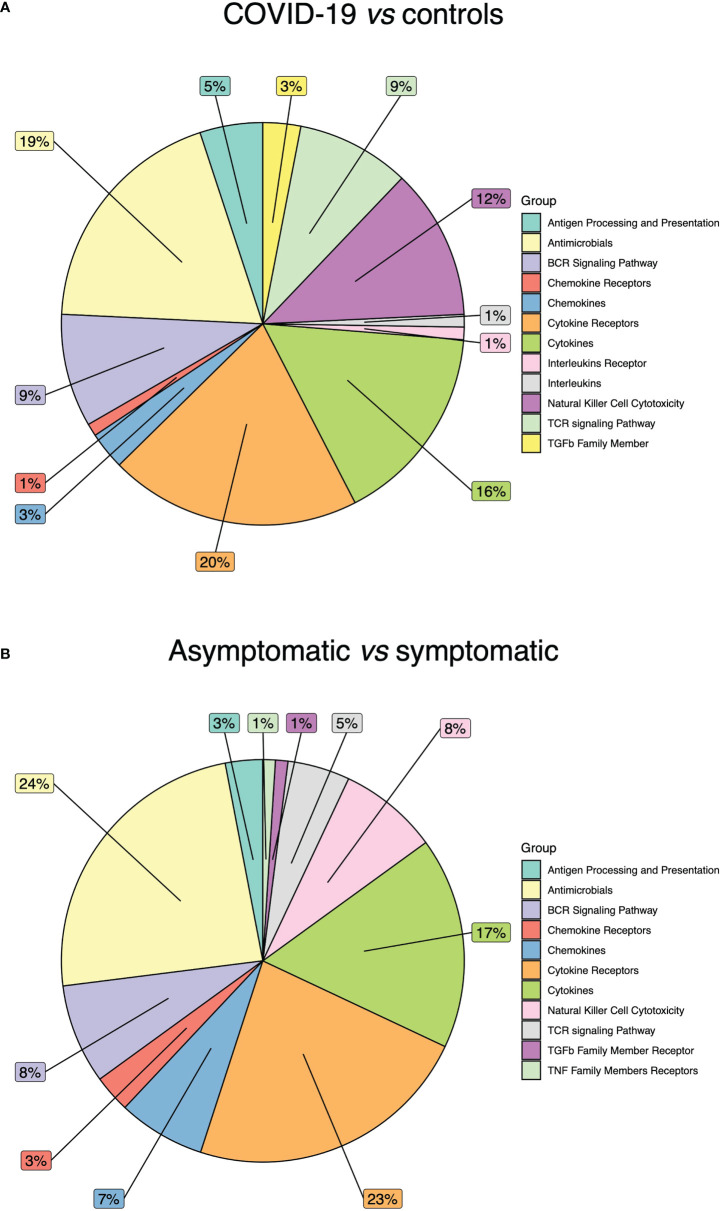
Immune related gene categories affected by DNA methylation alterations. **(A)** Pie chart showing the percentage of DNA methylation alterations in COVID-19 patients vs controls. **(B)** Pie chart showing the percentage of DNA methylation alterations in COVID-19 asymptomatic vs symptomatic patients.

The comparison between COVID-19 and control samples did not reveal alterations in these categories: Interferon Receptor, Interferons, TGFb Family Member Receptor, TNF Family Members and TNF Family Members Receptors. The most affected categories resulted: Cytokine Receptors (20%), Antimicrobials (19%), and Cytokines (16%) (**Figure 3A**). Of note, most differentially methylated CGIs were hypomethylated.

In the differential methylation analysis between asymptomatic and symptomatic patients, no alterations were detected in Interferon Receptor, Interferons, Interleukins Receptor, Interleukins, TGFb Family Member, TNF Family Member. The most affected categories were: Antimicrobials (24%), Cytokine Receptors (23%) and Cytokines (17%) (**Figure 3B**). The majority of DNA methylation alterations were hypermethylation events.

### 3.2 DNA methylation alteration of *HLA-C* in COVID-19 patients

It has been previously hypothesized that other coronaviruses such as SARS-CoV-1 and MERS-CoV affect the methylation pattern of *HLA* genes (3). Recently, a large epigenome-wide study of 407 COVID-19 patients has shown that methylation alteration of two CpG loci (cg08309069 and cg05030953) was negatively associated with the clinical severity of the disease.

**Figure 4:**
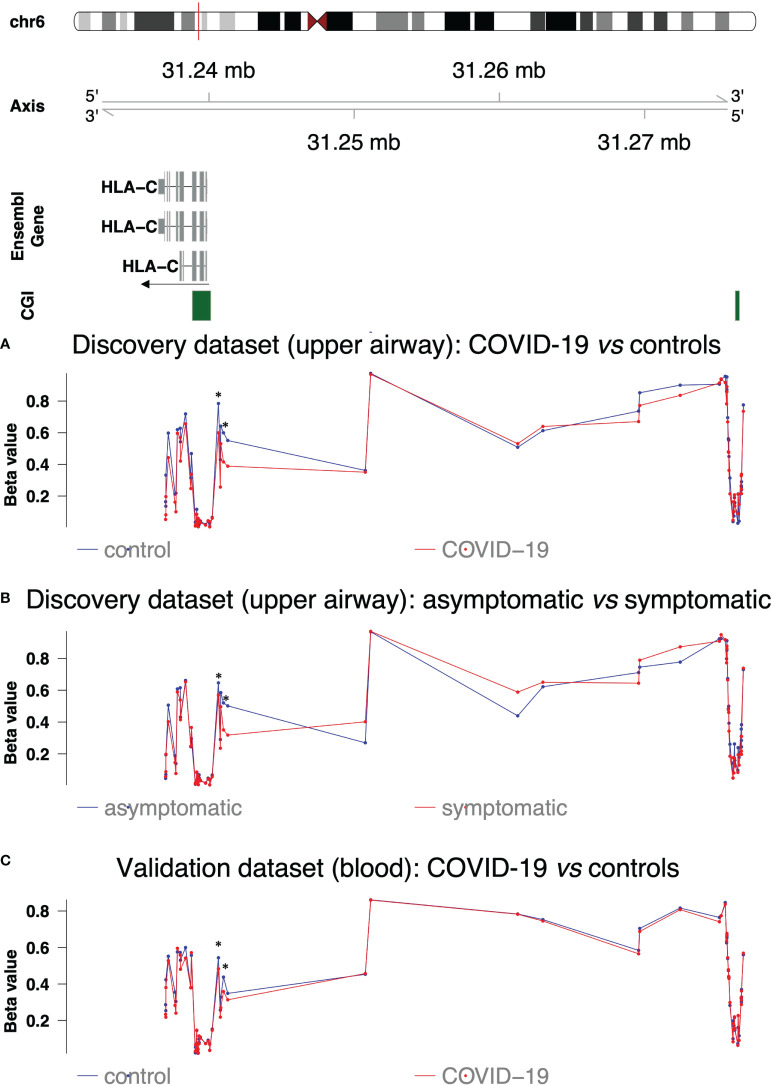
DNA methylation profile of *HLA-C* regions. The upper part shows *HLA-C* isoforms, CpG islands and their chromosomic localization. The arrow indicates direction of transcription **(A)** DNA methylation profile in the discovery dataset (upper airways) in COVID-19 patients (red line) and controls (blue line). **(B)** DNA methylation profile in the discovery dataset (upper airways) in COVID-19 symptomatic (red line) and asymptomatic patients (blue line). **(C)** DNA methylation profile in the validation dataset (blood) in COVID-19 patients (red line) and controls (blue line). Asterisks indicate the altered CpG sites identified in Castro de Moura et al. (13).

The DNA methylation analysis of immune-related genes has pointed out a CGI (chr6:31276242-31276526) associated with *HLA-C* that was hypermethylated (Δβ=0.05) in COVID-19 patients compared to controls (**Table 2**, **Figure 4A**). We carried out a comprehensive analysis of all CpG sites associated with *HLA-C* gene (**Table 2**, **Figures 4A, B**). It should be noted that from both the case-control and symptomatic-asymptomatic differential methylation analyses, no CGI associated with the transcription start site of the *HLA-A* and *HLA-B* genes was significantly altered (**Supplementary Figures 1A, B**).

**Table 2:** DNA methylation analysis in CpG sites associated with *HLA-C* gene in COVID-19 patients *vs* controls and asymptomatic *vs* symptomatic COVID-19 patients.

ID	Chromosome	Start	CGI	Relation to Island	Δβ COVID-19 patients *vs* controls	Δβ Asymptomatic *vs* symptomatic
cg09556042	chr6	31237013	chr6:31238852-31240120	N_Shore	-0.11	-0.01
cg11917734	chr6	31237029	chr6:31238852-31240120	N_Shore	-0.05	-0.02
cg11574174	chr6	31237034	chr6:31238852-31240120	N_Shore	-0.14	0.00
cg24710520	chr6	31237199	chr6:31238852-31240120	N_Shore	-0.16	0.10
cg01521131	chr6	31237664	chr6:31238852-31240120	N_Shore	-0.05	0.04
cg11867651	chr6	31237722	chr6:31238852-31240120	N_Shore	-0.12	0.06
cg11247343	chr6	31237824	chr6:31238852-31240120	N_Shore	-0.02	0.02
cg25149637	chr6	31238020	chr6:31238852-31240120	N_Shore	-0.06	0.08
cg13872627	chr6	31238036	chr6:31238852-31240120	N_Shore	-0.12	0.02
cg18511546	chr6	31238388	chr6:31238852-31240120	N_Shore	-0.06	0.01
cg26392102	chr6	31238751	chr6:31238852-31240120	N_Shore	-0.07	0.00
cg17096289	chr6	31238788	chr6:31238852-31240120	N_Shore	-0.13	-0.07
cg01230067	chr6	31239063	chr6:31238852-31240120	Island	-0.03	0.00
cg15397231	chr6	31239074	chr6:31238852-31240120	Island	-0.02	0.00
cg12965927	chr6	31239093	chr6:31238852-31240120	Island	0.00	0.00
cg18919024	chr6	31239133	chr6:31238852-31240120	Island	0.01	0.00
cg26128383	chr6	31239158	chr6:31238852-31240120	Island	-0.03	0.00
cg16181043	chr6	31239175	chr6:31238852-31240120	Island	0.00	0.00
cg18020334	chr6	31239243	chr6:31238852-31240120	Island	0.00	-0.01
cg18747378	chr6	31239247	chr6:31238852-31240120	Island	-0.01	0.00
cg24877963	chr6	31239266	chr6:31238852-31240120	Island	0.00	0.00
cg10409680	chr6	31239320	chr6:31238852-31240120	Island	0.03	0.03
cg17974398	chr6	31239324	chr6:31238852-31240120	Island	0.01	0.01
cg05619024	chr6	31239411	chr6:31238852-31240120	Island	-0.01	0.00
cg01006124	chr6	31239767	chr6:31238852-31240120	Island	0.00	0.00
cg06659144	chr6	31239939	chr6:31238852-31240120	Island	0.00	0.01
cg02827714	chr6	31240045	chr6:31238852-31240120	Island	0.01	0.00
cg27358585	chr6	31240050	chr6:31238852-31240120	Island	0.00	0.00
cg07505391	chr6	31240223	chr6:31238852-31240120	S_Shore	0.00	0.01
cg08309069*	chr6	31240651	chr6:31238852-31240120	S_Shore	-0.19	0.08
cg00620824	chr6	31240784	chr6:31238852-31240120	S_Shore	-0.17	0.05
cg12321669	chr6	31240814	chr6:31238852-31240120	S_Shore	-0.11	0.09
cg05030953*	chr6	31241000	chr6:31238852-31240120	S_Shore	-0.18	0.17
cg05338672	chr6	31241294	chr6:31238852-31240120	S_Shore	-0.16	0.18
cg13273236	chr6	31250765		OpenSea	-0.01	-0.13
cg04772476	chr6	31251137		OpenSea	-0.01	0.00
cg02119909	chr6	31261260		OpenSea	0.02	-0.15
cg16265787	chr6	31262996		OpenSea	0.03	-0.03
cg03813170	chr6	31269586		OpenSea	-0.07	0.07
cg25128801	chr6	31269662		OpenSea	-0.08	-0.04
cg01413481	chr6	31272449	chr6:31276241-31276526	N_Shelf	-0.06	-0.10
cg01830271	chr6	31275148	chr6:31276241-31276526	N_Shore	0.01	0.02
cg11794033	chr6	31275267	chr6:31276241-31276526	N_Shore	0.00	-0.02
cg03555881	chr6	31275551	chr6:31276241-31276526	N_Shore	-0.04	0.00
cg25107000	chr6	31275643	chr6:31276241-31276526	N_Shore	-0.08	0.03
cg14801238	chr6	31275664	chr6:31276241-31276526	N_Shore	-0.01	-0.01
cg26343358	chr6	31275666	chr6:31276241-31276526	N_Shore	-0.06	0.03
cg27071793	chr6	31275718	chr6:31276241-31276526	N_Shore	-0.03	0.01
cg27258561	chr6	31275767	chr6:31276241-31276526	N_Shore	-0.08	0.01
cg12076350	chr6	31275791	chr6:31276241-31276526	N_Shore	-0.08	0.04
cg02446475	chr6	31275807	chr6:31276241-31276526	N_Shore	-0.09	0.05
cg17250082	chr6	31275875	chr6:31276241-31276526	N_Shore	-0.10	0.08
cg12846737	chr6	31276088	chr6:31276241-31276526	N_Shore	0.01	0.00
cg23536255	chr6	31276105	chr6:31276241-31276526	N_Shore	0.07	-0.03
cg00648423	chr6	31276146	chr6:31276241-31276526	N_Shore	0.03	0.06
cg15225267	chr6	31276187	chr6:31276241-31276526	N_Shore	0.02	0.09
cg04953552	chr6	31276212	chr6:31276241-31276526	N_Shore	-0.02	0.03
cg02145102	chr6	31276418	chr6:31276241-31276526	Island	0.06	0.01
cg05419812	chr6	31276437	chr6:31276241-31276526	Island	0.00	0.04
cg06414921	chr6	31276504	chr6:31276241-31276526	Island	0.11	0.05
cg22993154	chr6	31276653	chr6:31276241-31276526	S_Shore	0.06	0.05
cg20412244	chr6	31276664	chr6:31276241-31276526	S_Shore	-0.01	0.07
cg13429614	chr6	31276667	chr6:31276241-31276526	S_Shore	-0.02	0.05
cg23366493	chr6	31276669	chr6:31276241-31276526	S_Shore	0.05	0.07
cg03634778	chr6	31276797	chr6:31276241-31276526	S_Shore	-0.04	-0.01

*CpG sites identified as altered in Castro de Moura et al. (13).

The results confirmed hypomethylation of the two CpG sites reported by Castro de Moura et al. (13) in symptomatic patients compared to asymptomatic ones but also in COVID-19 patients compared to controls (**Table 2** and **Figure 4B**). Hypomethylation was also extended to adjacent CpG sites in the S-shore of CGI located at chr6:31238852-31240120 (average Δβ= |0.14| and average Δβ= |0.10|, respectively in the differential methylation analyses between COVID-19 patients *vs* controls and asymptomatic *vs* symptomatic patients) (**Table 2** and **Figures 4A, B**). COVID-19 patients also displayed hypomethylation of the N-shore region of the same CGI (average Δβ= |0.09|), while the CGI itself was not differentially methylated (**Table 2** and **Figures 4A, B**).

Of note cg13273236 was not differentially methylated between COVID-19 patients and controls but hypermethylated in symptomatic patients (**Table 2** and **Figure 4A, B**) as well as the region between the S-shore of CGI at chr6:31238852-31240120 and the altered CGI at chr6:31276241-31276526.

#### 3.2.1 Validation in a publicly available dataset

In order to validate our results, we analysed GSE174818 dataset including methylation data from whole blood samples of 102 COVID-19 patients and 26 non-COVID-19 subjects (17). As evident from **Figure 4C**, we observed a similar methylation pattern throughout *HLA-C* gene although with less pronounced alterations.

### 3.3 *HLA-C* downregulation in COVID-19 patients

Transcript expression of *HLA-C* was evaluated in 61 COVID-19 patients, eight post-COVID-19 subjects and eight controls with no previous infection of SARS-CoV-2.

**Figure 5:**
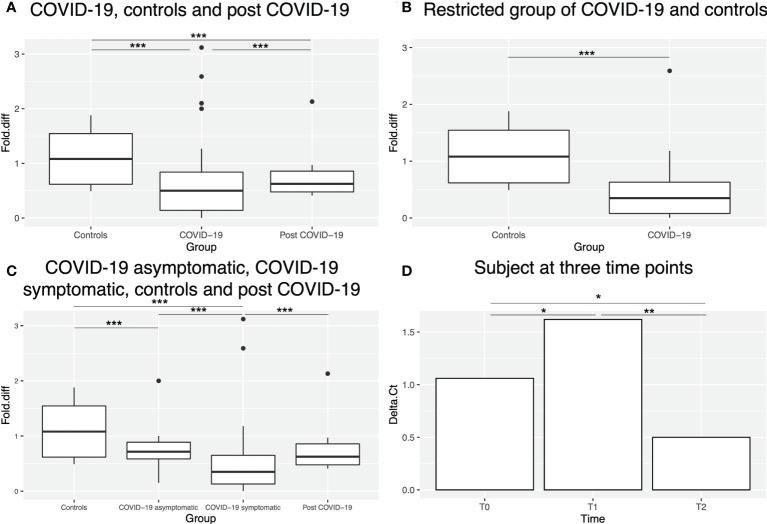
*HLA-C* expression in different sample groups. **(A)** Box plot showing *HLA-C* expression levels (as fold difference) in controls, COVID-19 and post-COVID-19 patients. Outliers are shown as dots outside the boxes. **(B)** Box plot showing *HLA-C* expression levels (as fold difference) in controls and the restricted group of COVID-19 patients (without potential confounding factors, see Materials and Methods). **(C)** Box plot showing *HLA-C* expression levels (as fold difference) in controls, asymptomatic, symptomatic and post-COVID-19 patients. **(D)** Bar plot showing *HLA-C* expression levels (as Delta Ct) in one subject at three time points. * indicates p-value <0.05, ** indicates p-value <0.01 and *** indicates p-value <0.001.

A statistically significant downregulation (p-value < 0.0001) was observed in COVID-19 patients compared to controls. Of note, post-COVID-19 subjects showed intermediate transcript levels between COVID-19 patients (p-value < 0.0001) and controls (p-value < 0.0001) (**Figure 5A**). In order to eliminate potential confounding factors that can affect *HLA-C* gene expression, we applied the same criteria of Castro de Moura et al. (13) as described in Materials and Methods. The reduction of *HLA-C* transcript levels was even more pronounced (p-value < 0.0001) in this restricted sample group (n=18) (**Figure 5B**).

We also investigated whether *HLA-C* expression could be correlated with COVID-19 severity. For this analysis, samples with missing clinical information were excluded. Indeed, the symptomatic group of patients (n=45) displayed statistically significant lower expression levels (p-value < 0.0001) than the asymptomatic (n=8) group (**Figure 5C**), that displayed a similar expression to post-COVID-19 subjects. Overall, both symptomatic and asymptomatic COVID-19 patients showed lower *HLA-C* expression than controls (p-value < 0.0001). Notably, among the symptomatic group, one patient, also analysed in the methylation study, was paucisymptomatic and actually displayed methylation and expression patterns more similar to asymptomatic patients and for this reason was finally considered in this group in the expression analysis.

Finally, we explored *HLA-C* expression in one subject at three different time points (**Figure 5D**): T0 before SARS-CoV-2 infection and T1 and T2 after COVID-19 recovery (both at a clinical point of view and negative to SARS-CoV-2 test), the last day of hydroxychloroquine treatment (T1) and 18 days after treatment (T2). A statistically significant decrease of *HLA-C* levels (p-value=0.027) was observed at T1 compared to T0, followed by statistically significant increase at T2 to levels even higher than T0 (p-value= 0.011).

### 3.4 Potential enhancers regulating *HLA-C* expression

**Figure 6:**
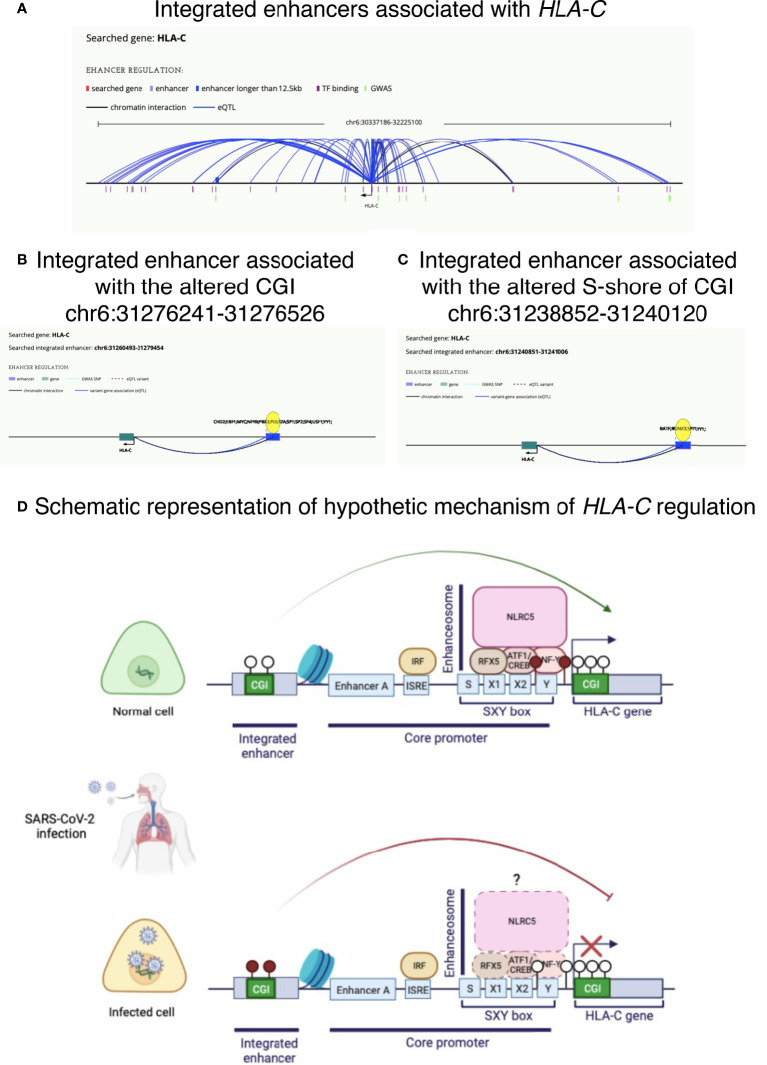
Potential altered mechanisms of *HLA-C* regulation in COVID-19 disease. **(A)** Integrated enhancers associated with *HLA-C*. **(B)** Integrated enhancer (chr6:31260493-31279454) associated with CGI chr6:31276241-31276526, found hypermethylated in COVID-19 patients. **(C)** Integrated enhancer (chr6:31240851-31241006) associated with S-shore region of CGI chr6:31238852-31240120, found hypomethylated in COVID-19 patients. **(D)** Schematic illustration of a possible mechanism of *HLA-C* downregulation observed in upper airway cells of COVID-19 patients. Lollipops are exemplificative representation of CpG sites, where empty and filled circles represent hypomethylated and hypermethylated CpG sites, respectively. Illustrations in panels **A–C** are from HACER database (http://bioinfo.vanderbilt.edu/AE/HACER/index.html); illustration in panel D was created with BioRender.com.

By consulting the HACER database, we found several integrated enhancers associated with this gene (**Figure 6A**). Interestingly, CGI chr6:31276241-31276526 (Δβ=0.05 in COVID-19 patients *vs* controls) overlaps with an integrated enhancer (chr6:31260493-31279454) (**Figure 6B**). Moreover, the analysis showed that the enhancers (AE_hg19_GM12878-ENCODE_504492 and AE_hg19_GM12878_21928) located at chr6:31275830-31276119 and chr6:31274921-31278342 (sub-regions of the integrated enhancer at chr6:31260493-31279454) in GM12878 (B-lymphocyte) cell line are bound by NFYB (among other transcription factors: EBF1, PBX3 and SP1) and regulates *HLA-C*, among others but the only *HLA*-class I gene targeted.

Moreover, part of the S-shore region of CGI chr6:31238852-31240120 altered in our work and in Castro de Moura et al. (13), belongs to an associated enhancer chr6:31240851-31241006, proximal to *HLA-C* gene (**Figure 6C**).

**Figure 6D** describes a hypothetic mechanism of *HLA-C* regulation by an enhancer.

## 4 Discussion

This study takes start from an EWAS analysis conducted on nasopharyngeal swab samples of a small group of 13 COVID-19 patients and three controls. As far as we know, this is the only methylome study performed to date on upper airway cells of COVID-19 patients, the first cells interested in the infection acting as APCs during respiratory diseases, since most of the previous research has been conducted on whole blood samples (13, 17, 27–29).

It is important to take into account that a mechanism observed in epithelial cells might be not evident in blood cells. In fact, Bortolotti and colleagues demonstrated that by setting up co-cultures of lung epithelial cells transfected with spike proteins and NK cells, intracellular expression of S1 SARS-CoV-2 protein in the epithelial cells reduces the activation of NK cells but this does not happen using lymphoblastoid cultures (30). The authors conclude that this phenomenon could explain the observation of a break in the interplay of lung epithelial cells and immune cells in SARS coronavirus patients, leading to an exhausted immune response (31).

The present research is based on the findings of a study conducted on epithelial cell cultures infected with various pathogenic viruses that has shown that MERS-CoV inhibits the antigen presentation by altering the epigenetic landscape of the host cell. In particular, the results suggested that DNA methylation, rather than histone modifications, plays a crucial role in MERS-CoV-mediated antagonism of antigen-presentation gene expression. Indeed, the authors observed, after infection, hypermethylation, and down-regulation, of genes associated with antigen presentation (3).

Despite the immense amount of scientific publications on SARS-CoV-2 infection, it is surprising that there has not been much attention on the expression levels of the genes of the HLA/MHC system, whose role in the immune response makes them very strong candidate genes. Nevertheless, the groups that have analyzed this aspect have generally found a reduced expression of HLA class I and II genes in agreement with our results (30, 32–35).

In fact, class I genes characterize the cell-mediated adaptive response, but MHC is also highly upregulated during the initial innate immune response.

Our work confirmed the presence of significant alterations in DNA methylation profiles between patients and controls and between symptomatic and asymptomatic patients. As in other published studies, although performed on different biological matrices (upper airway cells in the present study, blood in previous studies (13, 17)), the analysis of DNA methylation profiles reveals that most of the alterations are hypomethylation events and map at CGI associated with immune-related genes (17), in particular with those belonging to antimicrobials, cytokines and cytokines receptors categories.

Interestingly, in agreement with the results of Menachery et al. (3), we found a differentially methylated CGI (chr6:31276241-31276526) associated with *HLA-C*, a gene belonging to antigen processing and presentation category. Interestingly, Menachery et al. did not confirm *HLA-C* alteration neither at methylation nor gene expression level in cell lines infected by H1N1 influenza virus (3), as can also be observed in gene expression results obtained from a patient we examined, infected by H1N1 (data not shown). By analysing the methylation status of the entire *HLA-C* region, we focused our attention on an interesting overlap with the results obtained by Castro de Moura et al. (13) and also validated in Balnis et al. data (17), regarding two hypomethylated CpG loci, associated with the clinical severity of COVID-19, in an S-shore region of a CGI encompassing the first exons of *HLA-C* gene. Notably, our results showed that this association with the clinical severity is even accentuated and the alteration is also extended to other CpG sites in the S-shore and N-shore region of the same CGI (chr6:31238852-31240120), *per se* not altered. This observation may be due to the different type of cells analysed suggesting a more pronounced effect on respiratory cells, the first barrier of defense against respiratory infections. Of note, the region between the S-shore of this CGI and the next CGI displayed an extended hypermethylation in symptomatic COVID-19 patients compared to asymptomatic ones. Interestingly, as noted above, this last-mentioned CGI (chr6:31276241-31276526), upstream *HLA-C*, was hypermethylated in COVID-19 patients compared to controls. This observation is replicated by analyzing the data made available by Balnis and colleagues (17), obtained from a much larger cohort of patients.

The identification of a differential methylation pattern among patients with different prognosis, led us to investigate the expression levels of the gene. The consequent analysis of *HLA-C* expression by q-PCR actually showed a very statistically significant down-regulation of the gene in patients compared to controls, even more pronounced in the symptomatic ones, especially in those without other comorbidities, that could affect *HLA-C* expression. The levels seem to re-normalize after the viral clearance and disappearance of symptoms (post-COVID-19). Of note, it has been shown that ciliated cells from severe COVID-19 patients display a reduced overexpression of *HLA-C*, among other genes, compared with those from patients with moderate symptoms (36).

The association between the methylation status of the *HLA-C*-associated distal CGI and the region upstream *HLA-C* (S-shore region of CGI chr6:31238852-31240120) and the expression levels of the gene seems to fit perfectly with the hypothesis that this region represents enhancers for the *HLA-C* gene, as highlighted by the bioinformatic analysis and by the evidence that these regions are sensitive to DNase I and coincident with peaks of H3K27Ac, associated with the higher activation of transcription and therefore defined as an active enhancer mark. Importantly, it has been shown that DNA methylation may regulate the transcription of *HLA-A* locus (37), while it has been supposed that *HLA-B* and *HLA-C* expression is not regulated by DNA methylation since these alleles have been shown to be unmethylated (37). However, we found an altered DNA methylation pattern of this region in COVID-19 patients. Interestingly, the distal regulatory region is bound by NFYB, a transcription factor, part of the enhanceosome known to regulate *HLA* genes (38), and resulting to regulate *HLA-C* from the bioinformatic analysis. In fact, as known, while HLA class II molecules are expressed in specialized APCs, HLA class I molecules are ubiquitously expressed and different regulators are involved in their expression. NLRC5/CITA (NOD-like receptor family CARD domain containing 5/Class I TransActivator) is the MHC class I regulator in selected cell subset (38). However, this factor lacks a DNA binding domain and thus requires other factors, that collectively form the enhanceosome, to contact the MHC class I promoter region at the level of an SXY-module containing a S, X1, X2 and Y box (38, 39). Y box is bound by an NFY-complex consisting of NFYA, NFYB and NFYC subunits (40). Moreover, *HLA* class I genes are additionally regulated by distal enhancers other than core promoter elements. Interestingly, it has been shown that the mechanism by which SARS-CoV-2 can inhibit MHC class I pathway is the suppression both at transcriptional and functional level of NLRC5 in the lung and airway epithelial cells during infection, consequently interfering with the CD8 T cell action and leading to higher risk of exacerbation of viral loads and prolonged infection (34). However, as the authors explained the inhibitory effect of SARS-CoV-2-ORF6 on MHC class I suppression can be observed only under IFNγ treatment and thus cannot explain the downregulation observed in COVID-19 patients (34). Moreover, it is important to consider that *HLA-C*, in contrast to *HLA-A* and *HLA-B* do not present NF-kB binding sites and indeed its expression is weakly induced by inflammatory cytokines such as IFNγ (41). Therefore, it is plausible that DNA methylation of *HLA-C* regulatory region bound by the enhanceosome complex and the distal enhancer may be an additional mechanism contributing to HLA class I downregulation directly or by non-coding RNAs mapping on *HLA-C* regulatory region, as already suggested (42). In fact, it is known that DNA methylation of enhancers is associated with gene expression dysregulation (43). Moreover, also *NLRC5* expression may be dysregulated by other mechanisms such as promoter methylation, copy number alterations and genetic mutations and, as suggested, its expression levels may be associated with COVID-19 severity and mortality (34). For instance, HIV has been shown to alter the expression of *NLRC5* by regulating its DNA methylation pattern (44). Interestingly, we found that COVID-19 patients displayed altered methylation in *NLRC5*.

A further confirmation of the association between *HLA-C* expression levels and disease severity, is the case of a paucysymptomatic patient, although not statistically representative. The patient was initially classified in the symptomatic group, although presenting modest clinical signs. From the analysis of the methylation profile, the beta values of the loci examined were more similar to those found in non-symptomatic patients. *HLA-C* expression levels confirmed a phenotype more similar to asymptomatic patients than to symptomatic ones. Another interesting observation has emerged from the study of a subject followed since before the infection and at two different time points after the viral clearance. This subject showed a reduction of *HLA-C* expression levels at T1 and a recovery to the initial situation with even higher levels of expression.

The hypothesis above described, that DNA methylation, normally not used in cells to regulate *HLA-C* expression, could be instead exploited as a mechanism induced by SARS-CoV-2 to downregulate this locus, would be absolutely plausible considering the observations made in infections due to other viruses, such as HIV, particularly persistent and capable of evading the host’s immune response. Many pathogens evade CTLs by downregulating HLA molecules on infected cells. The strategy of a virus to induce HLA molecules downregulation, in particular HLA-C, is well known for retroviruses. For example, most primary HIV-1 clones downregulate *HLA-C*, reducing the ability of HLA-C restricted CTLs to suppress viral replication in CD4 + cells (15). The down-modulation of HLA-C can be also associated with its reduced binding to the respective inhibitory receptors (KIR) present on the surface of NK cells, dependent on both host genetics and the extent of virus-mediated HLA-C downregulation (45). Therefore, also host genetics can contribute to a different predisposition to viral infections and to a different scenario of responses to the pathogen (45). It should be pointed out that, although HLA-C is expressed at lower level at cell surface than the other HLA-class I molecules and therefore its role in the adaptive immune responses has been considered as marginal, it acts as a natural ligand for KIR that are able to recognize virtually all HLA-C allotypes (46). Therefore, as mentioned in the introduction, HLA-C represents a ligand for both T cell receptors and NK cell receptors (15, 16). These evolutionary characteristics conferring to *HLA-C* locus particular efficacy in exerting immuno pressure on viral infection, have probably made it a preferential target by viral mechanisms (15). It is therefore natural to consider the down-regulation of HLA-C as a mechanism to evade both CTL and NK mediated immune responses.

Once again, it is emblematic in this regard to observe HIV-1 infection, in which it has been shown that higher levels of HLA-C expression, regardless of specific allotypes, and specific peptides, are associated with better prognosis. This mechanism would be due at least in part to the consequent increase in the CTL-mediated response, thus exerting a higher immune pressure on the virus (47). Differences in expression even only twofold greater would improve CTL-mediated responses *in vivo* (48). Furthermore, HLA-C expression levels correlate inversely with viral load in patients not treated with anti-retroviral therapy (47).

HIV-1 modulates the HLA-C expression through the accessory protein Vpu, with different intensities by the various viral strains and adapting the down-modulation to the HLA-C genotype of the host (15, 49). As mentioned, contrary to HLA-A and HLA-B, virtually all HLA-C allotypes are recognized by a number of inhibitory and activating KIRs, making HLA-C a dominant ligand for the regulation of NK cell activity (50, 51). It has also been shown that KIR+ NK cells can recognize HIV-1-Vpu-mediated alterations of HLA-C expression (16, 45).

Consequently, it is not difficult to hypothesize that more “evolved” viruses aim at down-regulating HLA-C, which in turn is evolutionarily more diversified, therefore more capable of responding to the most varied types of infections, although lower expressed among *HLA* class I loci. It could be hypothesized that SARS-CoV-2 modulates the expression of HLA-C by means of an accessory protein similar to Vpu. In fact, among others, SARS-CoV-2 encodes a small transmembrane protein, called envelope (E), whose functions are not yet fully elucidated but which forms an ion channel that resembles, although different, viroporins such as Vpu (HIV) or M2 (influenza virus) (52).

However, as shown and discussed above, the results of our study also strongly suggest an epigenetic mechanism, *i.e.* inducing host DNA methylation alteration, by which SARS-CoV-2 could down-modulate HLA-C expression, as hypothesized for example for MERS-CoV (3).

A role for reduced KIR/HLA-C combination as risk factor for severe or fatal SARS-CoV-2 evolution has been demonstrated (in a cohort of patients coming from the same geographic area of this study); so a reduction of *HLA-C* expression such that we have found may reduce the activity of NK cells (in particular the memory-like NK) against the virus and thereby contribute to impaired viral clearance at early stages of infection (53).

From this evidence, it is clear that manipulation of the HLA class I presentation pathway through various mechanisms limiting their cell surface expression, which is shared by some other viruses (54) and also human coronaviruses (3), may represent a mechanism to escape/delay the early innate and adaptive immune response. This can reduce the efficacy of CD8+ T cells to recognize viral peptides presented by HLA class I molecules and thereby delay viral clearance, also not allowing a long memory of the infection to develop. By acting in this way, the virus would have shown an adaptation that makes it capable of maintaining its stay in the host population longer.

In conclusion, our results pointed out the reduction of *HLA-C* expression in COVID-19 patients, more pronounced in the severe cases, suggesting this molecule involved in antigen presentation as a potential prognostic marker and therapeutic target in RNA virus infections. Moreover, this discovery opens the possibility to design a vaccine conjugating SARS-CoV-2-specific antigen with an adjuvant that can stimulate the activation of T cells responsible for the immunological memory against the infection.

## Data availability statement

The data presented in the study are deposited in the MoBGE lab repository (Department of Biomedical Sciences, University of Cagliari), available here: https://drive.google.com/drive/folders/1C4EzXOhBqZW9XuoZep4nsUy1JfB8mPYu?usp=sharing.

## Ethics statement

The studies involving human participants were reviewed and approved by Ethics Committee of “ATS Sardegna” (224/2020/CE). The patients/participants provided their written informed consent to participate in this study.

## Author contributions

PZ and DF conceived the project. AC oversaw the management of COVID-19 patients. PZ coordinated the entire research project. PC, GA, GC, SD-G, DF and AC collected the samples and clinical information. PZ and LM purified and processed all DNA, RNA, cDNA samples for the whole-genome methylation assay and gene expression assay. EL performed the bioinformatics analyses of methylome data and functional annotation, performed gene expression assay. EL and PZ analysed gene expression data and interpreted results of all experimental procedures, wrote the original draft and final version of the manuscript. DF and HE made critical revision of the manuscript for important intellectual content. All authors critically reviewed and commented the manuscript. All authors contributed to the article and approved the submitted version.

## Funding

This work was supported by grants from Fondo per la Ricerca Locale (ex 60%), Università di Cagliari, to PZ and from Fondazione di Sardegna (CUP:F73C22001270007), to DF and PZ.

## Acknowledgments

We thank all COVID-19 patients and healthy participants for making this research possible. We wish to thank the healthcare workers from the sample collection centers for their kind collaboration and Dr. Sandra Orrù for advice and continuous support in this research.

## Conflict of interest

The authors declare that the research was conducted in the absence of any commercial or financial relationships that could be construed as a potential conflict of interest.

## Publisher’s note

All claims expressed in this article are solely those of the authors and do not necessarily represent those of their affiliated organizations, or those of the publisher, the editors and the reviewers. Any product that may be evaluated in this article, or claim that may be made by its manufacturer, is not guaranteed or endorsed by the publisher.
