# Supplementary material for: HLA-C dysregulation as a possible mechanism of immune evasion in SARS-CoV-2 and other RNA-virus infections
Source: Front Immunol. 2022 Oct 17;13:1011829. doi: 10.3389/fimmu.2022.1011829 (PMC9618630; doi:10.3389/fimmu.2022.1011829)
Supplement: Supplementary file 1 [file DataSheet_1.pdf]

Supplementary Material

1 Supplementary Tables

Supplementary Table 1. Clinicopathological characteristics of the COVID-19 patients and relevant data of healthy controls

ID	Clinical center	Status	Group	Gender	Age	Assay	Comorbidities	Clinical diagnosis
1	Santissima Trinità hospital	COVID-19	Symptomatic	M	68	Gene expression analysis	Rheumatoid arthritis, gouty arthritis, stenosis of the cardia in congestive gastropathy, osteoporosis, hepatic steatosis alcohol-related, grade II hemorrhoids	COVID pneumonia, intubated. Died one month after extubation from septic shock
2	Santissima Trinità hospital	COVID-19	Symptomatic	F	87	Genome-wide methylation analysis; Gene expression analysis	Hypercholesterolemia, atrial fibrillation	COVID pneumonia
3	Santissima Trinità hospital	COVID-19	Asymptomatic	F	26	Genome-wide methylation analysis; Gene	Acute hepatitis in protein malnutrition from anorexia nervosa	Asymptomatic

						expression analysis		
4	Santissima Trinità hospital	COVID-19	Symptomatic	M	49	Genome-wide methylation analysis; Gene expression analysis	Obesity, G6PD deficiency	COVID pneumonia, intubated
5	Santissima Trinità hospital	COVID-19	Symptomatic	M	46	Gene expression analysis	Psoriasis, G6PD deficiency	COVID pneumonia, non-invasive mechanical ventilation
6	Santissima Trinità hospital	COVID-19	Symptomatic	F	87	Genome-wide methylation analysis; Gene expression analysis	History of acute myocardial infarction, atrial fibrillation, hypothyroidism, diabetes mellitus	COVID pneumonia, low-flow oxygen. Died due to cachexia, cognitive impairment
7	Santissima Trinità hospital	COVID-19	Symptomatic	M	64	Gene expression analysis	Benign prostatic hypertrophy (BPH)	COVID pneumonia, non-invasive mechanical ventilation

8	Santissima Trinità hospital	COVID-19	Symptomatic	M	69	Genome-wide methylation analysis; Gene expression analysis	Autoimmune hepatitis / autoimmune cholangitis, history of HBV hepatitis, atrial fibrillation, gallbladder stones	Pneumonia, high flow oxygen
9	Santissima Trinità hospital	COVID-19	Symptomatic	F	58	Genome-wide methylation analysis; Gene expression analysis	Borderline personality disorder, post-traumatic stress disorder, fibromyalgia, overweight, G6PD deficiency	COVID pneumonia, high flow oxygen
10	Santissima Trinità hospital	COVID-19	Symptomatic	M	49	Genome-wide methylation analysis; Gene expression analysis	Down syndrome	COVID pneumonia, high flow oxygen
11	Santissima Trinità hospital	COVID-19	Asymptomatic	F	58	Genome-wide methylation analysis; Gene expression analysis	Overweight	Asymptomatic

12	Santissima Trinità hospital	COVID-19	Asymptomatic	M	96	Genome-wide methylation analysis; Gene expression analysis	Atrial fibrillation, chronic heart failure, atrerius hypertension, chronic obstructive pulmonary disease (COPD)	Bacterial pneumonia, asymptomatic
13	Santissima Trinità hospital	COVID-19	Paucisymptomatic	F	80	Genome-wide methylation analysis; Gene expression analysis	Outcomes of cerebral haemorrhage, ischemic and hypertensive heart disease	Paucisymptomatic
14	Santissima Trinità hospital	COVID-19	Symptomatic	M	35	Genome-wide methylation analysis; Gene expression analysis	None	COVID pneumonia, non-invasive mechanical ventilation
15	Santissima Trinità hospital	COVID-19	Asymptomatic	F	87	Genome-wide methylation analysis; Gene expression analysis	Arterial hypertension, reflux oesophagitis, senile dementia, osteoporosis	Asymptomatic

16	Santissima Trinità hospital	COVID-19	Asymptomatic	M	61	Genome-wide methylation analysis; Gene expression analysis	Ischemic heart disease	Asymptomatic, fever a month earlier
17	Santissima Trinità hospital	COVID-19	NA	F	34	Gene expression analysis	NA	NA
18	Santissima Trinità hospital	COVID-19	NA	M	49	Gene expression analysis	NA	NA
19	Santissima Trinità hospital	COVID-19	NA	F	75	Gene expression analysis	NA	NA
20	Santissima Trinità hospital	COVID-19	NA	M	66	Gene expression analysis	NA	NA
21	Santissima Trinità hospital	COVID-19	NA	M	71	Gene expression analysis	NA	NA
22	Santissima Trinità hospital	COVID-19	Symptomatic	M	77	Gene expression analysis	Lung cancer, bladder cancer	COVID pneumonia, no oxygen

23	Santissima Trinità hospital	COVID-19	Asymptomatic	F	66	Gene expression analysis	Amyloidosis, psoriasis, Hashimoto's hypothyroidism, osteoporosis, anemia, chronic kidney disease (CKD)	Asymptomatic
24	Santissima Trinità hospital	COVID-19	Symptomatic	F	61	Gene expression analysis	None	COVID pneumonia, high flow oxygen
25	Santissima Trinità hospital	COVID-19	Symptomatic	M	71	Gene expression analysis	Hypertension, benign prostatic hypertrophy (BPH)	COVID pneumonia, high flow oxygen
26	Santissima Trinità hospital	COVID-19	Symptomatic	M	83	Gene expression analysis	Acute kidney injury (AKI), diabetes, epilepsy, dementia, hyperuricemia, hypertension	COVID pneumonia, no oxygen
27	Santissima Trinità hospital	COVID-19	Symptomatic	F	56	Gene expression analysis	None	COVID pneumonia, low oxygen flows
28	Santissima Trinità hospital	COVID-19	Symptomatic	F	83	Gene expression analysis	Hypothyroidism, chronic obstructive pulmonary disease (COPD), hypertensive heart disease	COVID pneumonia, high flow oxygen
29	Santissima Trinità hospital	COVID-19	Symptomatic	M	47	Gene expression analysis	None	COVID pneumonia, low oxygen flows

30	Santissima Trinità hospital	COVID-19	Symptomatic	M	64	Gene expression analysis	Diabetes mellitus type I	COVID pneumonia, high flow oxygen
31	Santissima Trinità hospital	COVID-19	Symptomatic	F	39	Gene expression analysis	Pregnant 33 weeks	COVID pneumonia, no oxygen
32	Santissima Trinità hospital	COVID-19	Symptomatic	M	64	Gene expression analysis	Hypercholesterolemia, benign prostatic hypertrophy (BPH), column stabilization	COVID pneumonia, high flow oxygen, non invasive ventilation (NIV)
33	Santissima Trinità hospital	COVID-19	Symptomatic	M	52	Gene expression analysis	None	COVID pneumonia, high flow oxygen
34	Santissima Trinità hospital	COVID-19	Symptomatic	F	80	Gene expression analysis	Adnexectomy for ovarian cyst, thyroidectomy, cholecystectomy, hypercholesterolemia	COVID pneumonia, high flow oxygen, non invasive ventilation (NIV)
35	Santissima Trinità hospital	COVID-19	Symptomatic	F	59	Gene expression analysis	Hypertension, breast cancer	Pleural effusion (due to tumor), low oxygen flows

36	Santissima Trinità hospital	COVID-19	Symptomatic	M	65	Gene expression analysis	Arterial hypertension, obesity	COVID pneumonia, low oxygen flows
37	Santissima Trinità hospital	COVID-19	Symptomatic	M	76	Gene expression analysis	Ischemic heart disease, hypertension, subrenal aortic aneurysm	COVID pneumonia, low oxygen flows
38	Santissima Trinità hospital	COVID-19	Symptomatic	M	62	Gene expression analysis	Gastroesophageal reflux disease (GERD)	COVID pneumonia, low oxygen flows
39	Santissima Trinità hospital	COVID-19	Symptomatic	M	45	Gene expression analysis	Overweight	COVID pneumonia, high flow oxygen, non invasive ventilation (NIV)
40	Santissima Trinità hospital	COVID-19	Symptomatic	F	25	Gene expression analysis	Diabetes mellitus type I, obesity, Hashimoto's thyroiditis	Diabetic ketoacidosis
41	Santissima Trinità hospital	COVID-19	Symptomatic	F	64	Gene expression analysis	Gonarthrosis	COVID pneumonia, low oxygen flows
42	Santissima Trinità hospital	COVID-19	Symptomatic	M	57	Gene expression analysis	Hashimoto's thyroiditis, suspected chronic lymphocitic leukemia (CLL)	COVID pneumonia, low oxygen flows

43	Santissima Trinità hospital	COVID-19	NA	M	NA	Gene expression analysis	NA	NA
44	Santissima Trinità hospital	COVID-19	Symptomatic	M	72	Gene expression analysis	Obesity, hypertension, chronic obstructive pulmonary disease (COPD)	COVID pneumonia, high flow oxygen
45	Santissima Trinità hospital	COVID-19	Symptomatic	M	47	Gene expression analysis	NA	COVID pneumonia, high flow oxygen
46	Santissima Trinità hospital	COVID-19	Symptomatic	M	50	Gene expression analysis	Obese, hypertension	COVID pneumonia, high flow oxygen
47	Santissima Trinità hospital	COVID-19	Symptomatic	M	46	Gene expression analysis	Bipolar disorder	COVID pneumonia, low oxygen flows
48	Santissima Trinità hospital	COVID-19	Symptomatic	M	74	Gene expression analysis	Diabetes, hypertension, psoriasis	COVID pneumonia, low oxygen flows
49	Santissima Trinità hospital	COVID-19	Symptomatic	M	79	Gene expression analysis	Hypertensive heart disease, aortic insufficiency, benign prostatic hypertrophy (BPH), chronic kidney disease (CKD), hyperuricaemia, diverticulosis, previous stroke	COVID pneumonia, low oxygen flows

50	Santissima Trinità hospital	COVID-19	Symptomatic	M	49	Gene expression analysis	Hypertension	COVID pneumonia, high flow oxygen, non invasive ventilation (NIV)
51	Santissima Trinità hospital	COVID-19	Symptomatic	M	59	Gene expression analysis	NA	COVID pneumonia, high flow oxygen
52	Santissima Trinità hospital	COVID-19	Symptomatic	M	57	Gene expression analysis	Asthma, chronic gastritis	COVID pneumonia, high flow oxygen, non invasive ventilation (NIV)
53	Santissima Trinità hospital	COVID-19	Symptomatic	F	57	Gene expression analysis	Myasthenia gravis, previous thymoma, hypertension, dyspepsia	COVID pneumonia, high flow oxygen, non invasive ventilation (NIV)
54	Santissima Trinità hospital	COVID-19	Symptomatic	M	61	Gene expression analysis	NA	COVID pneumonia, low oxygen flows
55	Santissima Trinità hospital	COVID-19	Symptomatic	M	65	Gene expression analysis	NA	COVID pneumonia, high flow oxygen, non invasive ventilation (NIV)

56	Santissima Trinità hospital	COVID-19	Symptomatic	M	57	Gene expression analysis	NA	COVID pneumonia, low oxygen flows
57	Santissima Trinità hospital	COVID-19	NA	NA	NA	Gene expression analysis	NA	NA
58	Santissima Trinità hospital	COVID-19	NA	NA	NA	Gene expression analysis	NA	NA
59	University Hospital Policlinico Duilio Casula	COVID-19	Symptomatic	M	68	Gene expression analysis	Atrial fibrillation, cognitive deficity, arterial hypertension, adrenal adenoma	COVID pneumonia, lung score 16
60	University Hospital Policlinico Duilio Casula	COVID-19	Symptomatic	M	53	Gene expression analysis	Gastroesophageal reflux disease (GERD), ureteral lithiasis	Bilateral COVID pneumonia, pulmonary embolism
61	University Hospital Policlinico Duilio Casula	COVID-19	Asymptomatic	M	69	Gene expression analysis	Arterial hypertension, trachestomized laryngeal carcinoma, hypercholesterolemia, cerebral vasculopathy	Ischemic stroke

62 *	University Hospital Policlinico Duilio Casula	POST COVID-19; last day of treatment with hydroxychloroquine (T1)	Asymptomatic	M	39	Gene expression analysis	None	--
63	University Hospital Policlinico Duilio Casula	POST COVID-19	Asymptomatic	M	34	Gene expression analysis	None	--
64	University Hospital Policlinico Duilio Casula	POST COVID-19	Symptomatic	M	31	Gene expression analysis	None	--
65	University Hospital Policlinico Duilio Casula	POST COVID-19	Symptomatic	F	57	Gene expression analysis	None	--
66 *	University Hospital Policlinic	POST COVID-19; T2	Asymptomatic	M	39	Gene expression analysis	None	--

	o Duilio Casula							
67	University Hospital Policlinico Duilio Casula	POST COVID-19	Symptomatic	F	32	Gene expression analysis	Asthma	--
68	University Hospital Policlinico Duilio Casula	POST COVID-19	Symptomatic	F	43	Gene expression analysis	None	--
69	University Hospital Policlinico Duilio Casula	POST COVID-19	Symptomatic	F	47	Gene expression analysis	None	--
70*	University Hospital Policlinico Duilio Casula	Control	--	M	39	Genome-wide methylation analysis; Gene expression analysis	None	--

71	University Hospital Policlinico Duilio Casula	Control	--	F	30	Gene expression analysis	None	--
72	University Hospital Policlinico Duilio Casula	Control	--	M	52	Genome-wide methylation analysis; Gene expression analysis	Asthma in therapy	--
73	University Hospital Policlinico Duilio Casula	Control	--	F	49	Gene expression analysis	None	--
74	University Hospital Policlinico Duilio Casula	Control	--	M	31	Genome-wide methylation analysis; Gene expression analysis	Allergic rhinitis	--

75	University Hospital Policlinico Duilio Casula	Control	--	F	29	Gene expression analysis	None	--
76	University Hospital Policlinico Duilio Casula	Control	--	F	31	Gene expression analysis	None	--
77	University Hospital Policlinico Duilio Casula	Control	--	M	31	Gene expression analysis	None	--

* indicates the same subject at three different time points

2 Supplementary Figures

A. *HLA-A*

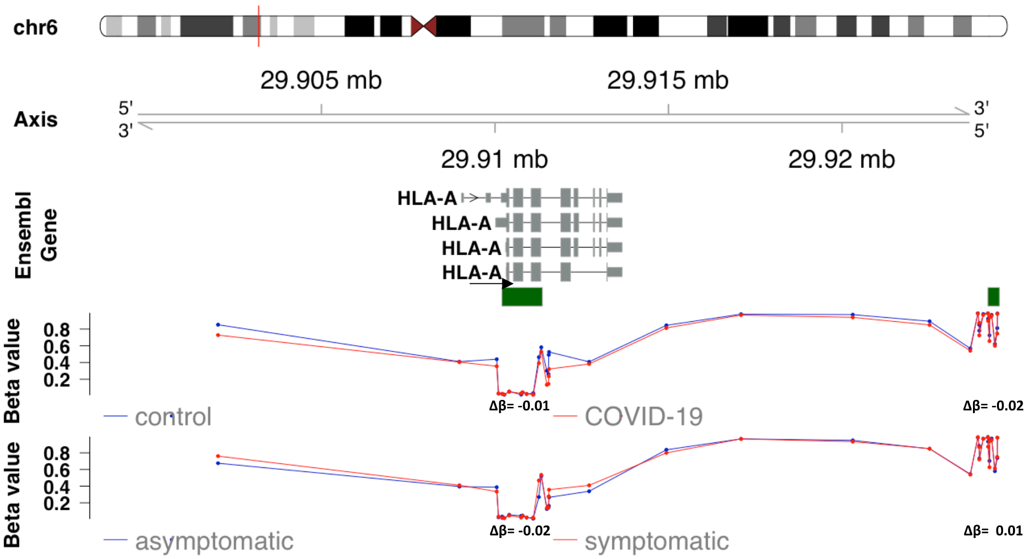

A. *HLA-B*

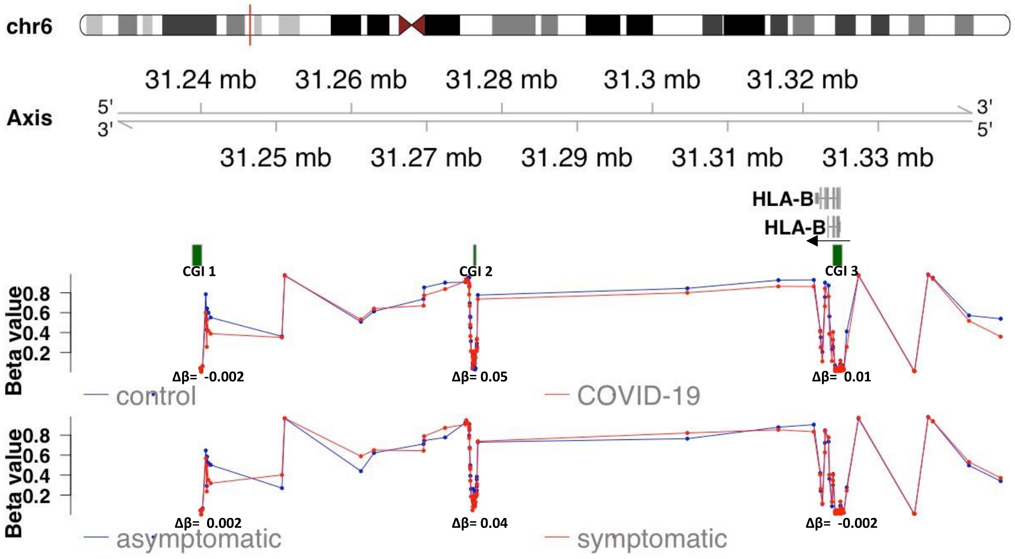

Supplementary Figure 1. DNA methylation profile of *HLA-A* and *HLA-B* regions. (A) *HLA-A* region methylation profile in the discovery dataset (upper airways) in COVID-19 patients (red line)

and controls (blue line) and in COVID-19 symptomatic (red line) and asymptomatic patients (blue line). The upper part shows *HLA-A* isoforms, CpG islands and their chromosomic localization. The arrow indicates direction of transcription. CGI average Δβ in case/control and asymptomatic/symptomatic is reported (B) *HLA-B* region methylation profile in the discovery dataset (upper airways) in COVID-19 patients (red line) and controls (blue line) and in COVID-19 symptomatic (red line) and asymptomatic patients (blue line). The upper part shows *HLA-B* isoforms, CpG islands and their chromosomic localization. The arrow indicates direction of transcription. CGI average Δβ in case/control and asymptomatic/symptomatic is reported

Note: CGI 1 and CGI 2 are shown for their proximity to *HLA-B* genomic coordinates but they are associated to *HLA-C* considering their proximity to the transcription start site and direction of transcription.
